# Tracing contacts of TB patients in Malaysia: costs and practicality

**DOI:** 10.1186/2193-1801-1-40

**Published:** 2012-10-24

**Authors:** Muhammad Atif, Syed Azhar Syed Sulaiman, Asrul Akmal Shafie, Irfhan Ali, Muhammad Asif

**Affiliations:** 1 Discipline of Clinical Pharmacy, School of Pharmaceutical Sciences, Universiti Sains Malaysia, Penang, Malaysia; 2 Department of Pharmacy, The Islamia University of Bahawalpur, Punjab, Pakistan; 3 Discipline of Social and Administrative Pharmacy, School of Pharmaceutical Sciences, Universiti Sains Malaysia, Penang, Malaysia; 4 Respiratory Department, Penang General Hospital, Penang, Malaysia; 5 Department of Pharmacology, School of Pharmaceutical Sciences, Universiti Sains Malaysia, Penang, Malaysia

**Keywords:** Tuberculosis, Tuberculin skin test, X-ray, Activity based costing, Contact tracing, Penang General Hospital

## Abstract

Tuberculin skin testing (TST) and chest X-ray are the conventional methods used for tracing suspected tuberculosis (TB) patients. The purpose of the study was to calculate the cost incurred by Penang General Hospital on performing one contact tracing procedure using an activity based costing approach. Contact tracing records (including the demographic profile of contacts and outcome of the contact tracing procedure) from March 2010 until February 2011 were retrospectively obtained from the TB contact tracing record book. The human resource cost was calculated by multiplying the mean time spent (in minutes) by employees doing a specific activity by their per-minute salaries. The costs of consumables, Purified Protein Derivative vials and clinical equipment were obtained from the procurement section of the Pharmacy and Radiology Departments. The cost of the building was calculated by multiplying the area of space used by the facility with the unit cost of the public building department. Straight-line deprecation with a discount rate of 3% was assumed for the calculation of equivalent annual costs for the building and machines. Out of 1024 contact tracing procedures, TST was positive (≥10 mm) in 38 suspects. However, chemoprophylaxis was started in none. Yield of contact tracing (active tuberculosis) was as low as 0.5%. The total unit cost of chest X-ray and TST was MYR 9.23 (2.90 USD) & MYR 11.80 (USD 3.70), respectively. The total cost incurred on a single contact tracing procedure was MYR 21.03 (USD 6.60). Our findings suggest that the yield of contact tracing was very low which may be attributed to an inappropriate prioritization process. TST may be replaced with more accurate and specific methods (interferon gamma release assay) in highly prioritized contacts; or TST-positive contacts should be administered 6H therapy (provided that the chest radiography excludes TB) in accordance with standard protocols. The unit cost of contact tracing can be significantly reduced if radiological examination is done only in TST or IRGA positive contacts.

## Introduction

Tuberculosis (TB) is a major global health problem with an annual incidence rate of 9 million cases worldwide. It is the largest single infectious cause of mortality among young individuals and adults in the world, accounting for approximately two million deaths every year (World Health Organization 2000). Similar to other developing countries, TB is still a public health problem in Malaysia despite the preventive and control measures taken. The incidence rate of TB in Malaysia has been at around 85 to 82 per 100,000 populations in the last five years. However, the absolute number of new cases has been increasing from about 15,000 in 2002, up to 20,000 in 2011 (Ministry of Health 2012). Pertaining to its highly contagious nature, inadequate investigation of the contacts of index case may be one of reasons for its re-emergence.

Fundamental objectives of TB control are to detect the disease as early as possible, and to make sure that those diagnosed complete their treatment and are cured. In the mid-1990s, the Directly Observed Treatment Short course (DOTS) strategy was adopted as method for tuberculosis control (World Health Organization 2009). Estimates suggest that the introduction of DOTS could halve the current potential national economic loss from TB (World Health Organization 2000).

In this era of economic decline, health care managers need to use the most cost effective tracing and treatment measures to halt the progression of the disease. Contact tracing is the process of identifying the relevant contacts of a person with an infectious disease (the index patient) and ensuring they are aware of their exposure (Australasian Society for HIV Medicine 2010). The World Health Organization (WHO), the International Union Against Tuberculosis and Lung Disease (IUATLD) and the International Standards for Tuberculosis Care (ISTC) recommend as a minimum:

-screening households and close contacts of smear positive pulmonary tuberculosis cases to detect new TB cases;
-for children under five years of age and for all people with HIV without symptoms suggestive of TB, providing isoniazid preventive therapy (IPT) [Fair et al. 2009; World Health Organization 2008; World Health Organization 2006).

The scope of contact tracing differs between settings. Tuberculin Skin Testing (TST) and chest X-ray are the most commonly employed contact tracing procedures (Australasian Society for HIV Medicine 2010; Centers of Disease Control and Prevention 2005; Richard and Kerri 2010); however TST is comparatively less cost-effective than radiological examination (Schwartzam and Menzies 2000). Guidelines (Centers of Disease Control and Prevention 2005; Richard and Kerri 2010;Joint Tuberculosis Committee of the British Thoracic Society 2000) on contact tracing recommends TST to all HIV-negative household members and/or close contacts of infectious TB cases who are five years of age or older, and have had active TB excluded. Furthermore, guidelines recommend administering IPT in contacts with positive TST. A study from Germany has showed that chemoprevention by Isoniazid is a cost-effective approach for reducing the burden of tuberculosis (Diel et al. 2005).

The basic underlying condition of any efficient allocation of resources is knowing the financial cost of the disease. Without analyzing the costs it is impossible to contemplate or improve the efficiency of disease-control projects. In particular, the ongoing reform and decentralization processes in the health care systems of developing countries require precise cost information (Su et al. 2007). On the most part, health care organizations use cost accounting to the estimate unit cost of their services that could help plan a realistic budget and price for the service (Sumeet et al. 2010). Conventional costing system utilizes a single, volume-based cost driver and has therefore, failed to cope with the challenges of rapidly evolving processes and product technologies.It has been well established that conventional accounting method overestimates high volume products, while underestimating low volume products. This gives an incorrect relationship between production and costs (Ames and Hlavacek 1990).

To date, most of hospital managers rely upon information from conventional accounting systems that was designed when competition was local rather than global, and when the rapidity and quality of an item or service was less decisive for success (Cohen et al. 2000). However, many companies have found a better cost-accounting method: activity based costing (ABC) (Kaplan 1992). ABC is a dynamic approach to determine unit costs by identifying principal activities, linking indirect costs to products or services through time allocation, and other tracing methods (Waters et al. 2001; Brimson 1991; Copper 1989). The result is a more accurate estimate of the real unit costs. Many hospitals and health organizations in United States have explored and used ABC to improve resource management (Waters et al. 2001).

We conducted our study at the Respiratory Clinic of Penang General Hospital (PGH), Penang, Malaysia to determine the cost of single contact investigation including chest X-ray and TST. We also aimed to compare the practices and results of contact tracing in the current setting with some of the established protocols. Existing literature suggests that data on the costs and practicality of contact tracing of people associated with the index cases is either scarce or unavailable. We expect that the findings of our study could have a significant impact on the principles and practices of contact tracing in the local setting, and may help other National Tuberculosis Programs (NTPs) to review their procedures with similar statistics.

## Methods

### Setting and study duration

The Respiratory Clinic of PGH has a Directly Observed Treatment Short Course (DOTS) facility and staff responsible for the treatment of registered TB patients. DOTS staff are also responsible for contact investigation of TB associates. Contact tracing records of TB associates were explored from the contact tracing log book from 1st March 2010 to 28th February 2011 to evaluate costs and practicality of the procedure in local setting.

The Radiology Department of PGH has a designated facility and staff for performing chest X-rays (labeled as room 2). Data consisted of 31431 radiological examinations performed during the period of January 1st to December 31st, 2010.

Total costs for chest X-ray included the human resource costs, capital costs, consumable costs and overhead costs. Cost components for TST included the human resource and consumable costs.

### Contact tracing procedure at study site

A contact tracing procedure at the Respiratory Clinic of PGH starts with the notification of the index case. After notification, index case details (notification form) are sent to the District Health Center (DHC). The Health Inspector at DHC visits and conducts an interview with the index case within a week of notification. In the case of the index case not being available at home, proxy interviews are conducted. After interviewing, details of contacts (including household contacts, friends, colleagues, class fellows etc.) are recorded on a specific form. All the listed contacts are asked (either face to face or by telephone) to visit the Respiratory Clinic at PGH for screening for active or latent TB. After finishing the interview, one copy of the finalized list of contacts is given to the responsible staff at the Respiratory Clinic of PGH. Once the contact arrives at the Respiratory Clinic, DOTS staff responsible for contact tracing performs a TST. The contact is advised to re-visit the DOTS center between the next 48–72 h (i.e. on the third day). At the same time, the contact is advised to undergo a radiological examination (chest X-ray) at the Radiology Department of PGH. Developed X-ray film is dispatched to the Respiratory Department. A medical doctor at the Respiratory Department examines the chest X-ray and informs the staff nurse at the DOTS clinic, whether or not the X-ray findings are indicative of possible TB. In these cases, the contact is further investigated using TB-specific laboratory tests.

It is the responsibility of the Respiratory Clinic staff to inform the health inspector if any contact fails to report on the expected dates. In such case, the health inspector re-communicates with the missing contacts and further advices them for contact tracing activity.

### Human resource cost

Interviews with key DOTS and radiology personnel were conducted to identify the principal activities for TST and chest X-rays. This was followed by determination of the time taken to complete each activity using a stopwatch (LeBaron et al. 1999). The duration was captured 15 times each for three alternate days and summarized as the mean, median, the 25th and, the 75th quartiles for each activity (LeBaron et al. 1999). The personnel time for each of the employees involved was valued according to the pay scale of the Federal Civil Services Officers under the System of Remuneration Malaysia (Jabatan Perkhidmatan Awam Malaysia 2011). Prior to the valuation, these salaries were converted into the salary per minute (MYR/min) by assuming a daily working time of 8 h and a monthly working time of 20 days. The cost of each employee per single activity was obtained by multiplying the mean time (minutes) spent by that employee doing a specific activity by his/her salary per minute (MYR/min). Finally, the total manpower cost incurred per service was the sum of the human resource costs of all activities involved producing the service.

Moreover, human resource idle time cost for chest X-rays was calculated by multiplying mean idle time between two consecutive activities of each employee with their salary per minute. Idle time cost for a medical doctor was not calculated as he/she shared other responsibilities at the Respiratory Clinic of PGH. Similarly, idle time cost for TST was not calculated because staff nurse performing this activity was also sharing other activities at DOTS center. Idle time cost was not included in final cost.

### Capital costs

For the unit cost calculation of a chest X-ray, the costs of the equipment used were obtained from the procurement section of the Radiology Department. The costs of the building were calculated by multiplying the area size for the service with the unit cost of public building (MYR 85/ft^2^). Area size of the chest X-ray facility was also provided by the public building department of Penang General Hospital. The useful life was assumed to be five years for clinical equipment and 30 years for building (Meigs and Meigs 1996). Moreover, straight-line deprecation with a discount rate of 3% was assumed. At the end of the asset’s useful life, the resale value was considered to be 10% of the initial costs (Drummond et al. 1997). The equivalent annual cost for each was calculated based on the following equations:

The unit asset cost was obtained by dividing the equivalent annual cost of each asset by the total number of X-ray films exposed in the year 2010.

The cost of the building and machines was not calculated for TST as the DOTS facility was used for certain other TB related activities.

### Consumable costs

The consumables for a chest X-ray included X-ray film, fixer and developer reagents and, the envelope for the developed film. The quantity and costs of each X-ray film and the envelope were obtained from the procurement section of the Radiology Department. The total cost of the fixer and developer reagents per X-ray was obtained by dividing the total cost of reagents in one year by the number of tests in year 2010.

Tuberculin PPD RT 23 SST (1.5 mL vial) and 1 cc syringe were the only consumables for TST. Tuberculin PPD RT 23 SST vial (1.5 mL) is recommended for use in 10 individuals. However, based on the number of contacts visiting DOTS during its labeled stability (24 h), it was used in only eight contacts, on average.

### Electricity costs

For the unit cost calculation of a chest X-ray, annual electric power consumption (kW/h) for an X-ray machine, day light developer machine and tubes were calculated separately and then multiplied by the unit price of one kW/h (MYR 0.312/kWh) (Tenaga Nasional Berhad 2011) to get the annual electric cost for each electrical appliance. The annual electric cost for each appliance was divided by the number of X-rays done in 2010 to get the cost per X-ray film.

Electricity cost was not calculated for TST as the DOTS facility was used for other TB management related activities.

### Data analysis

The socio-demographic and clinical profiles of the study participants were presented in terms of frequency and percentage. Staff activity was recorded in minutes. All costs were reported in MYR followed by conversion to US Dollar at an exchange rate of USD1 = MYR3.19.

## Results

Table 1 describes the socio-demographic and clinical characteristics of TB contacts.

**Table 1:** **Socio-demographic and clinical characteristics of TB contacts**

Socio-demographic characteristics	N (%)
**Total sample (TST and X-ray)**	1024 (100)
**Sputum positive Index**	239
**Sputum negative and Extra pulmonary TB index**	85
**Gender**	
Male	436 (42.6)
Female	588 (57.4)
**Ethnicity**	
Malay	407 (39.7)
Chinese	494 (48.3)
Tamil	56 (5.5)
Others	67 (6.5)
**TST reading**	
<10 mm	547 (53.4)
≥10 mm	38 (3.8)
No records	439 (42.8)
**Chest X-ray findings suggestive of TB**	No record available
**Isoniazid started (latent TB infection)**	0 (0)
**Notified as Confirmed case of TB (active case detection)**	5 (0.5)

### Human resource costs

Total human resources cost for a single contact tracing procedure was MYR 7.29 excluding idle time cost (Table 2). Six distinct activities to produce an X-ray film were identified which includes receiving and allocating specific number to a patient (attendant 1); registering the patient in the log book (clerk); preparing and exposing the patient to X-rays and developing the film in daylight machine (radiographer 1); labeling the film envelope and validating/sorting films to meet the standard criteria (radiographer 2); dispatching films to the respective wards/clinics (attendant 2); and examination of the chest X-ray by a medical doctor. Radiographer 1 and 2 were the designated staff for chest X-ray, while the clerk, attendant 1, attendant 2 and, the doctor were the shared human resources.

**Table 2:** **Human resource (HR) cost for a single contract tracing procedure**

Staff	Activities	Mean time (minutes)	Median time (25th,75th) (minutes)	Salary per minute (MYR)	Cost per unit (MYR)	Percentage from total HR cost	Idle time (seconds)	Idle time cost (MYR)
**Chest X-ray**								
Attendant 1	Receiving and allocating number	1.15	0.98 (0.89,1.3)	0.141	0.162	3.1	8	0.018
Clerk	Patient registration	0.74	0.72 (0.55,1.0)	0.145	0.107	2.2	5	0.012
Radiographer 1	Preparing and exposing patient	3.43	3.0 (2.3,4.6)	0.211	0.723	13.9	12	0.042
Radiographer 2	Labeling and validating film	1.79	1.4 (1.2,2.0)	0.169	0.302	5.8	107	0.299
Attendant 2	Dispatching films	1.36	1.3 (1.3,1.4)	0.141	0.190	3.7	6	0.138
Medical Doctor	Chest X-ray Screening	7.9	7.6 (6.3,8.6)	0.470	3.58	71.3	-	-
Human resource cost per chest X-ray					**5.06**	Idle time cost per chest X -ray		**0.51**
**Tuberculin Skin testing (TST)**								
Staff nurse	Recording contact details, injecting PPD, reading and recording result	14.9	14.7 (13.3,16.3)	0.150	**2.23**	100	-	**-**
**Total Human resource cost for contact tracing (Chest-X ray + TST) = MYR7.29**								

For TST, a staff nurse at the Respiratory Clinic of PGH performed the following duties: a) counseling the patient about the advantages of TST and, recording individual details on a contact card and in the contact tracing log book; b) intradermal injection of Purified Protein Derivative (PPD); c) counseling the patient to report at the Respiratory Clinic between 48–72 h; and d) recording the TST result on contact card and in the contact tracing log book.

### Capital costs

Capital costs for TST were not calculated as the facility was shared by other activities/services. However capital costs for a chest X-ray included the cost of X-ray machine (Philips™), cost of the daylight developer equipment (Agfa, Compact EOS™) and cost of the building (designed facility for chest X-ray labeled as room number 2). Cost (per film) of X-ray equipment was the highest (MYR 1.63), followed by the daylight developer equipment (MYR 0.32) and the building (MYR 0.03). Total capital cost per chest X-ray film was MYR 1.98 (USD 0.62). Table 3 shows the equivalent annual costs and unit costs of assets.

**Table 3:** **Equivalent annual cost and unit costs of assets**

Asset	Equivalent annual cost (MYR)	No of test done in 2010	Unit cost (MYR)	Percentage
X-ray machine	51208.3	31431	1.63	82.3
Daylight developer	44825.5	140973	0.32	16.2
Building	1010.4	31431	0.03	1.5
**Total unit asset cost**			**1.98**	**100**

### Consumable costs

The total consumable cost for a single contact tracing procedure was MYR 11.72. The total cost of consumables for chest X-ray was MYR 2.15. Consumables for chest X-ray included developer and fixer reagents (MYR 0.24 per X-ray film), envelop (MYR 0.26 per X-ray film), and X-ray film (MYR 1.65 per X-ray film).

Total cost of consumables for TST was MYR 9.57. This included Tuberculin PPD RT 23 SST (MYR 9.32 per TST) and a 1 cc syringe (MYR 0.25 per TST). Cost of a 1.5 mL vial of Tuberculin PPD RT 23 SST (sufficient for 10 applications) was MYR 74.58. However, the unit cost was based on its use on an average of eight contacts per day.

### Overhead costs (electricity)

Overhead costs for TST were not calculated as the facility was shared by other activities/services. The electricity costs (per film) for X-ray equipment, daylight developer equipment and tubes were MYR 0.002, MYR 0.031 and MYR 0.01, respectively. Total electricity cost per chest X-ray film was MYR 0.043.

### Total cost per contact tracing procedure

The total cost for a single contact tracing procedure (chest X-ray and TST) was MYR 21.03 (Table 4).

**Table 4:** **Overall cost per one chest X-ray film and Tuberculin Skin Testing**

Resources	Cost (MYR)	Cost (USD)*	Percentage (%)
**Chest X-ray**			
Human resources cost	5.06	1.59	24.1
Capital costs	1.98	0.62	9.4
Consumable costs	2.15	0.68	10.3
Overhead costs	0.04	0.01	0.2
**Total cost**	**9.23**	**2.90**	**-**
**Tuberculin Skin testing (TST)**			
Human resources cost	2.23	0.70	10.6
Consumable costs	9.57	3.0	45.4
**Total cost**	**11.8**	**3.70**	**-**
**Total cost per one contact tracing (Chest-X ray + TST)**	**21.03**	**6.60**	**100**

*1USD = 3.19MYR (Available from http://www.xe.com/ucc/convert/?Amount=1&From=USD&To=MYR).

## Discussion

Prevention of TB infection in healthy individuals is one of the major targets set by the World Health Organization (WHO). In majority of developed and developing countries, associates of newly diagnosed TB patients should be investigated for active and latent TB infection (Underwood et al. 2003). However, competing demands restrict the resources that can be allocated to contact-investigation of TB associates. Therefore, TB health care managers must decide which contact investigations should be assigned high priority (Centers of Disease Control and Prevention 2005). The criteria to prioritize contacts as high, moderate and low is listed in various guidelines (Centers of Disease Control and Prevention 2005; Richard and Kerri 2010; National Isntitute for Heatlh and Clinical Excellence 2006). However, Malaysian guidelines (Malaysian Thoracic Society 2012) do not comment on this aspect. Our study findings have indicated that prioritization of the contacts by the health inspector was done in an arbitrary way without following written procedures that could lead to wastage of valuable resources. Center for Disease Control and Prevention in the United States America has described detailed criteria to prioritize contacts (Centers of Disease Control and Prevention 2005). They have also suggested that the prioritization of contacts has a favorable impact on efficiency of the contact investigation procedure. Looking at the current medical records of index cases (Wilce et al. 2002; Centers of Disease Control and Prevention Centers for Disease Control and Prevention 2003), determining the infectious period (Reichler et al. 2002; California Department of Health Services 2005; Centers of Disease Control and Prevention 1994) and interviewing the patients (Centers of Disease Control and Prevention Centers for Disease Control and Prevention 1994; Centers of Disease Control and Prevention 2010) are components of the identification and prioritization procedure. Proxy interviews and field investigations (Bates et al. 1965; Bock et al. 1998) are sometimes beneficial. Similarly, anatomical site of disease, results of sputum bacteriology, radiographic findings, age, and sociability of index cases are some key indicators that can facilitate the decision to initiate contact investigation among contacts. Competency of the contact investigation staff is a key to the success of the process.

Different NTPs employ various contact tracing procedures depending upon the availability of resources. However, TST and chest X-ray are the most commonly employed investigations (World Health Organization World Health Organization 2009; Centers of Disease Control and Prevention Centers for Disease Control and Prevention 2005; Underwood et al. 2003; National Institute of Health and Clinical Excellence 2006). Many countries including the United States and the United Kingdom limit their contact investigation to high and medium priority contacts which are classified based upon available guidelines (Centers of Disease Control and Prevention 2005; National Institute of Health and Clinical Excellence 2006). Once the contacts are labeled as high and medium priority, investigations including TST and chest X-ray should be initiated as soon as possible. As per our study findings, health inspectors usually counseled the contacts to visit the DOTS center within two weeks irrespective of the priority assigned to them. Contrary to this, United States guidelines stress to conduct the test within seven and fourteen days for high and medium priority contacts, respectively. However a window period of 8–10 weeks is recommended for previously sensitized individuals (Centers of Disease Control and Prevention 2005).

Six months of Isoniazid (6 H) preventive therapy is recommended in contacts with positive TST results provided that the chest radiograph does not show evidence of TB (World Health Organization 2009; Centers of Disease Control and Prevention 2005). Different guidelines suggest different cut-off points for a positive TST (World Health Organization 2009; Richard and Kerri 2010). The WHO and the National Institute for Health and Clinical Excellence (NICE) in the United Kingdom follow a cut-off point of ≥10 mm for a positive TST (World Health Organization 2009; National Institute of Health and Clinical Excellence 2006). However, the United States guidelines follow a cut-off point of ≥5 mm to initiate 6 H (Centers of Disease Control and Prevention 2005). According to our study findings, TST was ≥10 mm in 38 subjects; however 6 H was started in none. This seems to be a clear deviation from the standard protocols. Guidelines on contact tracing from Pacific Island countries suggest not administering TST to contacts unless the NTP can offer 6 H therapy to TST-positive contacts and monitor this treatment (Richard and Kerri Richard and Kerri 2010). The United States guidelines on contact tracing suggest employing chest X-ray only when TST is positive. Taking this recommendation into account, this strategy could perhaps save valuable resources, especially in our setting. Contrary to this, other guidelines (Richard and Kerri 2010; National Institute of Health and Clinical Excellence National Institute for Health and Clinical Excellence 2006), including the United Kingdom guidelines (Joint Tuberculosis Committee on the British Thoracic Society Joint Tuberculosis Committee of the British Thoracic Society 2000), suggest investigating through both TST and chest X-ray. NICE guidelines further advice an interferon-gamma test if TST is positive (National Institute of Health and Clinical Excellence National Institute for Health and Clinical Excellence 2006).

It has long been known however, that the TST is far from ideal, due to low sensitivity and specificity (particularly from significant cross-reactivity to Bacille Calmette-Guérin [BCG]) and numerous operational drawbacks. QuantiFERON-TB Gold (QFT-G; Cellestis, Carnegy, Australia) and T-SPOT.TB (Oxford Immunotec, Oxford, UK) are most the most recent advances for the detection of latent TB infection (Arend et al. 2007; Zellweger Zellweger et al. 2005). Both of these tests are included in the United Kingdom guidelines, recommending a two-stage strategy of TST testing followed by an Interferon Gamma Release Assay (IRGA) to confirm a positive TST result. However, there are no studies that have demonstrated the validity of this approach (National Collaborating Center for Chronic Conditions 2006). Recent United States guidelines issued by CDC recommend that QFT-G may be used in all circumstances in which the TST is currently used (Mazurel et al. Mazurek et al. 2005).

A recent meta-analysis (Morrison et al. 2008) has shown that the yield of TB contact tracing (active case) in low and middle income countries is 6.5% (aged >15 years). Our findings show an active case detection of 0.5% (active tuberculosis) which is quite low. This great difference may be associated with the different criteria to prioritize contacts for investigation. However, future studies are required to confirm the reasons for such a large gap.

Based on our findings, and from recommendations made by various guidelines, Malaysian protocols used to investigate the contacts of TB patients need appropriate revisions. TST may be either replaced with more specific methods, or TST-positive individuals must be given chemoprophylaxis using 6 H or Isoniazid (H) and Rifampicin (R) for 3 months (3HR). Opting 3HR preventive therapy could reduce the chances of non-compliance. Authors would also suggest that contact investigation should only be limited to individuals classified as high and medium priority. This measure could perhaps lead to better yield of contact tracing.

One of major strengths of our study is that we have employed the ABC approach to estimate the cost a of single contact investigation. ABC has been successfully implemented in various manufacturing and service organizations. However, there have been only few reports on implementation of the ABC in health care (Laurila et al. 2000). To date no study has reported the cost of contact tracing using ABC. Our findings on the cost of contact tracing have strong potential to help Malaysian health care managers to take corrective actions for process improvement. For example our findings have shown that radiographer 2 remained idle for 1.78 min (107 s), which was almost equal to his activity time (1.79 min). This clearly suggests that radiographer 2 can share another similar activity in the Radiology Department, thereby, saving some human resource cost. Similarly, our findings would allow health care managers to evaluate the resources utilized versus the benefits achieved. Our findings would also allow policymakers to revise their decision-making tree to investigate a TB patient associate, while keeping in mind the possible unit cost of the investigation.

## Conclusion

Our findings have suggested that the yield of contact tracing was very low, which might be attributed to inappropriate prioritization process. Our findings also indicated that chemoprophylaxis was not initiated in TST-positive contacts. Therefore, either TST may be replaced with more accurate and specific methods (IRGA) in highly prioritized contacts, or TST-positive contacts must be administered 6 H therapy in accordance with standard protocols. The unit cost of contact tracing can be significantly reduced if radiological examination is done only in TST- or IRGA-positive contacts.

### Study limitation

While calculating the unit cost of a contact investigation, the cost of the health inspector was excluded as it was not possible for him to recall the amount of time and resources (telephone calls, personal visits etc.) he spent on each contact.
